# The use of YouTube in developing the speaking skills of Jordanian EFL university students

**DOI:** 10.1016/j.heliyon.2021.e07543

**Published:** 2021-07-12

**Authors:** Hadeel A. Saed, Ahmad S. Haider, Saleh Al-Salman, Riyad F. Hussein

**Affiliations:** Applied Science Private University, Department of English Language and Translation, Amman, 11192, Jordan

**Keywords:** YouTube, Speaking, EFL, ESL, Jordan, Experimental group

## Abstract

This study examines the effectiveness of using YouTube videos in teaching the speaking skills among English as a Foreign Language (EFL) students in Jordan. The study sample comprised 80 students attending Oral Skills classes in the English Language and Literature Department at a private university in Jordan. The participants were equally divided into a control group and an experimental group of 40 students each. The experimental group was taught through the use of YouTube videos, while the control group was taught the speaking skills using the traditional approach. A pre-test and a post-test were administered to the two groups. Four TEFL experts were asked to rate the participants’ performance using the IELTS speaking band descriptors, which consist of four main categories: fluency & coherence, lexical resource, grammatical range & accuracy, and pronunciation. The findings showed that the performance of the two groups was improved. However, compared to the traditional group, the experimental group demonstrated a relatively better improvement. The results also showed significant progress in the speaking performance of the students subjected to the YouTube experiment. Of all the four constructs under investigation, pronunciation and fluency & coherence were the most noticeably advanced in the performance of the YouTube experimental group. The present study recommends that YouTube videos be embedded into the EFL classroom to improve students' speaking skills.

## 1 Introduction

Technology has invaded every aspect of our lives, the most vital of which is education, where the teaching-learning process is the prime target. Most modern-day classrooms are innovative in nature, being equipped with a smart-board or a data-show projector. Traditional classroom, with a standard curriculum given by a teacher face-to-face, where traditional paper-and-pencil tests are administered, is no longer the favored option. We live in a world dominated by state-of-the-art digital technology featuring smartphones, iPads, and YouTube videos supported by highly sophisticated ICT applications in all walks of life, top among which is education. To cope with this sweeping wave of change in educational technology, educators, curriculum designers, and experts in EFL pedagogy have been cognizant of the pressing need for a parallel shift in teaching and testing strategies at all levels of education. Proponents of this shift have become more affirmative in their demands in the wake of the COVID-19 pandemic and its huge challenges, forcing a global shift in the teaching-learning paradigm towards online learning (Almahasees et al., 2021; Haider and Al-Salman, 2020b). This emerging situation has brought about new realities that made education technology a necessity, which will perhaps set the stage for blended and/or full-fledged online learning much sooner than ever (Dhawan, 2020; Li and Lalani, 2020; Martin et al., 2020).

This study investigates the impact of YouTube on developing students’ speaking skills. For this purpose, a control group and an experimental group were selected. The control group was taught the speaking skills through the use of traditional activities, while the latter group was subjected to learning the same skills through YouTube. A pre-test was administered to both groups, and the scores of the two groups were compared. A post-test was then administered, which showed that the test scores in all the speaking skill descriptors were moderately higher for the experimental group, which can be attributed to the treatment of YouTube use.

In Jordan, with the availability of Internet-supported digital technology, more audiovisual learning resources have been in-store, with the YouTube videos dominating the scene. However, one deterring factor is the lack of proper training for some instructors who are still lagging behind and cannot cope with the latest advances in digital learning and assessment technology. Nowadays, most students have computer know-how that some teachers may be unfamiliar with, which has produced a rift between them and their teachers. Prensky (2001) argues that the digital gap between generations resulted in group of digital immigrant teachers who cannot cope with the sophisticated technical knowledge of their students in the EFL classrooms. Luckily, however, such sporadic cases can be remedied and should not interfere with wide-scale implantation, which turned to be more urgent than any time before in the wake of the coronavirus crisis.

In the last two decades, YouTube has become a rich and relevant platform for social media users because it hosts diverse views and activities. YouTube videos can enhance the English teaching and learning process. Its content can be used by EFL students to develop language skills especially speaking and listening comprehension.

Given the above, we can say that the use of YouTube in language learning has been researched in many countries, for example, Iran (Akbari and Razavi, 2016), Indonesia (Meinawati et al., 2020; Pratama et al., 2020; Tristiana and Swondo, 2020), Saudi Arabia (Almurashi, 2016; Arroyyani, 2018; Dhawan, 2020), Sudan (Omer, 2017), and Algeria (Djahida, 2017). However, only a few studies, to the best of the researchers' knowledge, have been conducted regarding the use of YouTube as a source of learning in the Jordanian context, especially for developing the speaking skill, a major productive skill for effective communication. Therefore, it is expected that the present study will help bridge this gap by investigating the effect of using YouTube videos in improving Jordanian university students’ speaking skills. Furthermore, the need for conducting such a study in the Jordanian context has become so pressing due to the shift to the online teaching-learning model brought by the COVID-19 pandemic (Al-Salman and Haider, 2021b).

In sum, the primary objective of the current research is to assess the impact of using YouTube videos on improving students' speaking skills, which are regarded as key elements in determining proficiency in the language. Thus, the present study addresses the following questions:

1 Is there significant progress and improvement in the speaking performance of the students subjected to YouTube compared to those who are taught the traditional way?
2 If so, which of the following subscales, fluency & coherence, lexical resource, grammatical range and accuracy, and pronunciation, were most noticeably advanced in the YouTube experimental group's performance?

## 2 Review of related literature

The use of YouTube videos in the foreign language classroom has catered to meeting students' needs and teachers' orientation. This new portal provides fast and instant videos of instructional and entertaining materials worldwide. The majority of these videos fulfill a two-fold function; first, they are fun to watch, and second, they are an effective learning medium that students enjoy. Consequently, students have a real opportunity to engage meaningfully in learning the target language. What is striking about the use of YouTube is that students are learning without knowing that they are learning.

Numerous studies have been conducted regarding the use of videos to improve language skills. These studies have brought enormous benefits to the field of teaching in general and EFL classes in particular. Various scholars anticipated the application of all forms of electronic technology to improve the learning process. YouTube offers a vast range of useful content for teaching English. Through interacting in a fun experience, students are stimulated, and their insecurities about learning a second language are reduced. This approach offers an excellent opportunity for teachers to help their students excel and progress. Compared to traditional teaching methods, YouTube videos are more realistic, practical, and comprehensive (Almurashi, 2016).

Interestingly, a large proportion of YouTube content is educational, where a vast range of videos present valuable information in lots of fields such as medicine, science, politics, history, and so on. YouTube is also a significant source of relevant social material that can encourage students to engage with popular culture in different countries. A recent study conducted by Syafiq et al. (2021) showed that YouTube videos have contributed significantly to enhancing not only the students’ speaking skills but also other English language proficiency components including grammatical structures, lexicon, fluency, and content.

There are several benefits associated with using video clips to teach a foreign language. Nasution (2019) believes that YouTube is an invaluable instructional tool for language teachers. It is a very effective medium for developing language skills through vocabulary building, presenting topics for debate and dialogue, which represent all varieties of English used in the EFL classroom. According to Cakir (2006), language instructors find the video content both interesting and motivating as it creates a real, contextualized, and authentic teaching-learning environment.

Cakir (2006) also reported that videos provide authentic language inputs that produce a constructive language acquisition atmosphere. In these kinds of authentic environments, students encounter real objects, individuals, and circumstances. Akbari and Razavi (2016) examined the use of authentic resources in EFL classrooms. Their research findings showed that the use of authentic materials has a positive impact on students' performance. Moreover, authentic resources reduce the complexities of teaching in the EFL classroom.

Anggraeni (2012) and Pratiwi (2011) noted that the use of videos helps students organize ideas, choose the right words, create coherent sentences, and use the correct mechanics of writing (punctuation and spelling). As YouTube is one of the most highly used portals globally, it could be employed in the EFL classrooms to improve students' proficiency in the English language, especially their speaking and listening skills. This may create a new dimension for a more engaging and fruitful educational environment (Alkathiri, 2019). The audiovisual feature of YouTube videos makes them appealing to students as they teach and educate in real-life situations and contexts. This is driven by the fact that visuals offer access to real language use in authentic environments and social contexts. Although many suggestions have been made to enhance the educational process inside the classroom, such as websites, blogs, video-sharing websites, iTunes, and Vimeo, YouTube seems to be the most widely used and has become extremely popular, especially among young adults (Alimemaj, 2010). Moreover, Silviyanti (2014) reported that using YouTube in the EFL classroom seems compelling, encouraging, and beneficial, where students appear to be enthusiastic and willing to watch different videos, then practice pronunciation and speaking in the same way as native speakers.

Another advantage of using YouTube in classrooms is that it is cost-effective. There is no limit on the amount that it can be used online, and watching videos is free of charge. Yagci (2014) highlighted these benefits and noted that YouTube is a global gateway that can be accessed anywhere and anytime. Furthermore, according to Bueno Alastuey (2011), YouTube videos play a significant role in enabling learners to speak and communicate with their teachers and classmates and understand their surroundings. Watkins and Wilkins (2011) pointed out that YouTube is a powerful teaching aid which boosts learning both inside and outside the classroom. It provides a multi-media platform for promoting all language learning skills, especially listening and speaking, together with enriching students' vocabulary in different domains and cultural backgrounds. The results of a study conducted by Jati et al. (2019) showed that using YouTube content has improved the students’ speaking skill considerably in three areas: fluency, accuracy and overall performance.

Different scholars reported similar findings on the effectiveness of YouTube videos in improving students’ speaking skills (see Albahlal, 2019; Arroyyani, 2018; Pratama et al., 2020; Setiawan and Wiedarti, 2020; Tristiana and Swondo, 2020). Qomar (2016) examined the use of YouTube in enhancing students' speaking performance and found that YouTube could improve students' speaking skills. The study showed that students used good intonation and stress when pronouncing sentences, correct grammatical structures, and an accurate and sufficient choice of words. They were also able to initiate a conversation without hesitation, unnecessary silences, or repetition of words. Similarly, Wagner (2007) recommended using videos to teach speaking skills because videos enable students to remember the information, excel in pronunciation, understand what is said through the graphics and illustrations used in videos, and speak fluently and freely. The research findings reported by Kurniawan (2019, p. 324) showed that students cling to the YouTube under the premise that it is an effective tool for improving their speaking skill. In a similar vein, research findings by Meinawati et al. (2020) showed that watching YouTube videos helped students speak more fluently and confidently.

Riswandi (2016) examined how YouTube videos can help improve students' speaking skills. The study was conducted on seventh-grade students at one of the junior high schools in Indonesia. The findings showed that the use of YouTube resulted in clear progress in the students' speaking skills, especially vocabulary, grammar, pronunciation, and fluency. In the same vein, Omer (2017) investigated the effectiveness of using YouTube videos in improving EFL learners' listening and speaking skills. A questionnaire was used to collect data from a sample of 30 s-year undergraduate students of English. The results showed that YouTube improved students’ listening and speaking skills and encouraged them to communicate verbally in English. Furthermore, YouTube acquainted students with native English speakers' culture, which, in turn, strengthened their grasp of the language.

Djahida (2017) examined the impact of educational YouTube videos in enhancing EFL learners' speaking ability. The participants included ten teachers who taught the ‘Oral Expression’ Module and 60 Master's students from the English Department at Biskra University in Algeria. The researcher found that the teachers and students looked positively at using YouTube videos to improve speaking skills. Baniabdelrahman (2013) investigated the effect of using online-shared oral diaries on Saudi first-year university students. Two male and two female students participated in the study. The experimental and control groups each consisted of one female and one male participants. The researcher developed a speech proficiency test and instructed both groups to complete a pre-test before conducting the study and a post-test at the end of the intervention. The results showed that the test score was in favor of the experimental group.

With this background in mind, and the voluminous research on how the YouTube videos have positively impacted and contributed to promoting the EFL teaching/learning paradigm, the current study sets to investigate the effect of the YouTube tool in enhancing the speaking skill of Jordanian EFL university students. Informed by the research findings of the reviewed literature, the present study will be more enlightened about the pathway it will take in determining the research methodology, analysis, discussion of results, and conclusions that will enlighten all stakeholders in the EFL domain.

## 3 Methodology

### 3.1 Study sample and ethics approval standards

The participants in this study consisted of 80 students enrolled in four English Oral Skills classes offered by the Department of English Language and Literature at a private university in Jordan. Each class comprised 20 students. The course is usually given to students in their third year based on the Department's advisory plan. The four classes were equally divided into two groups, experimental and control.

The 80 participants were divided equally between the control group and the experimental group. There were twenty-four female participants in the control group compared to twenty-eight in the experimental group. This reflects the high female-male student ratio in the Department. All participants are junior-level students who have completed 75–90 credit hours of the BA program in English. They are approximately in the age group of 20–23.

The current study's ethical approval was obtained from the Deanship of Scientific Research at the Applied Science Private University in Jordan with the approval number (FAS/2020–2021/202). Also, a written informed consent was obtained from all subjects before the study was conducted.

### 3.2 Research design

A pre-test was administered to both groups to measure their performance in the speaking skills. For assessment purposes, four TEFL experts were assigned to rate the participants' performance using the IELTS speaking band descriptors, consisting of four main categories: fluency & coherence, lexical resource, grammatical range & accuracy, and pronunciation. Each category consisted of nine bands, where zero characterizes students not attending the exam, while 9 characterizes participants with highly developed sub-skills in all four categories (see Appendix 1). After the implementation of the experimental program, a post-test was given to both groups to see whether or not the students’ achievements in the speaking skill have improved.

With regard to the teaching program, the traditional group was taught the speaking content traditionally, while the experimental group was taught the same skill using YouTube. After a period of 16 weeks in which students were subjected to a 3-hour teaching per week, a post-test was administered to both groups. Again, the four TEFL experts were asked to assess the students’ performance in the above-mentioned speaking subskills. To make sure that the evaluation of the four examiners is consistent, we conducted an interrater reliability analysis of the four examiners for each construct as Table 1 shows.

**Table 1:** Interrater reliability analysis for the four examiners for each construct.

Construct	Measure of Agreement: Kappa	*p-value*
Fluency and Coherence	0.894	0.000
Lexical Resource	0.955	0.000
Grammatical Range and Accuracy	0.949	0.000
Pronunciation	0.961	0.000

The results of the interrater analysis for the four constructs, namely, fluency & coherence, lexical resource, grammatical range & accuracy, and pronunciation were Kappa values 0.894, 0.955, 0.949 and 0.961, respectively, with p < 0.001 for each construct. These values represent outstanding levels of agreement (Landis and Koch, 1977).

Concerning the experimental group, the following procedures were followed before introducing the YouTube videos to the students. The researchers first watched the target videos to judge their suitability in terms of form and content. Then the suitable ones were chosen, and students were required to watch them at home before discussing them in the classroom. Subsequent to this, the teacher was asked to prepare a set of guiding questions. After a detailed description of the goals of these videos and their value in developing the speaking skill, students were required to do a number of tasks over a period of four months, starting from the simple to the more complex and from yes/no questions to Wh-questions. Besides, students were taught summary activities and techniques and how to summarize the video content. They were also required to present oral reports guided by a set of questions given to classmates in advance.

The traditional approach was implemented by using the activities given in the prescribed textbook over a period of one semester (16 weeks). These activities focus more on matters related to form, such as grammar, vocabulary, and pronunciation. Real communication, such as conveying or obtaining information, is relegated to a minor position. So the majority of activities here revolve around drilling, where the teacher or a student asks a question, and another classmate gives an answer. This question-answer practice or session is structured and anticipated, and in most cases, there is only one correct and pre-determined answer. The focus, therefore, is on the form of questions, and little attention is given to the communicative function and interaction. So information gap is lacking where students are required to accomplish a task such as conveying meaning in English, obtaining the required information, or expressing their viewpoints. In other activities such as summarization, where students summarize a text or passage, the same pattern prevails where the teacher pays attention to pronunciation, word usage, and correct grammar rather than main ideas or meaning.

Two male instructors were assigned to teach, one for the experimental group and another for the control group. Both instructors had an MA in TESOL and Linguistics and more than eight years of relevant teaching experience.

### 3.3 Validity and reliability

To ensure that the four IELTS speaking band descriptors are internally reliable and distinguishable from one another, the researchers applied Cronbach's alpha for each subscale on a sample of 20 respondents as Table 2 shows (Cronbach, 1951).

**Table 2:** Reliability analysis through cronbach alpha results.

Group	No. of Items	Cronbach's Alpha
Control Group	4	0.967
Experimental Group	4	0.971
All Variables	8	0.916

Table 2 shows a high level of reliability and internal consistency. A reliability coefficient of 0.70 or higher is considered “acceptable” in social science research (Nunnally, 1978).

Based on the results above, a test of normality was conducted using Skewness and Kurtosis, as Table 3 shows.

**Table 3:** Results of normality test.

Group	Test	Skewness	Kurtosis
Control Group	Pre-Test	0.346	-0.130
	Post-Test	0.424	-0.313
Experimental Group	Pre-Test	0.061	-0.392
	Post-Test	0.372	0.572

Table 3 contains the Skewness and Kurtosis values. Data is considered to be close to the normal distribution if it lies (-3 and +3) (George and Mallery, 2003). Based on the obtained values, it is clear that the data is normally distributed as they lie between the assigned range.

## 4 Results

We first checked whether or not there is significant progress in the speaking performance of the students who were subjected to the two teaching methods, i.e., we compared the students' performance before and after taking the Oral Skills course. In Table 4, the “M (SE)” column provides the Mean of the students’ scores, the standard error for that Mean and Standard Deviation, and paired sample t-test to test the significant progress in the speaking performance of the students who were subjected to the two teaching methods.

**Table 4:** Descriptive statistics and paired sample T-Test.

Nu	Group		M (SE)	SD	Mean Difference	T value
					M (SE)	
1	Control Group	Pre-Test	4.86 (0.16)	0.9805	-0.19 (0.04)	-4.392∗∗∗
		Post-Test	5.05 (0.16)	0.9987		
2	Experimental Group	Pre-Test	4.95 (0.15)	0.9793	-0.55 (0.05)	-10.356∗∗∗
		Post-Test	5.50 (0.16)	0.9935		

(SE) Standard error of mean, (SD) Standard Deviation, ∗∗∗p < 0.001, ∗∗p < 0.01, ∗p < 0.05.

There appears to be progress in the students' scores after being subjected to a 16-week teaching course. This is evident in the difference in the students' scores in the pre-test and post-test. Table 4 shows a statistically significant improvement in the students’ scores among the two groups, where the t values were less than -2, and the *p-value* was less than 0.001 (Everitt and Skrondal, 2002).

After confirming that there is a statistically significant difference in the performance of the two groups before and after taking the course, we checked which group has improved more. In other words, we tested the differences between the control group and the experimental group. To do so, we conducted an Independent Sample T-Test, as Table 5 shows.

**Table 5:** Independent sample T-Test.

Group	Mean (SE)	Mean Difference	T value
		Mean (SE)	
Control Group	4.91 (0.11)	-0.37 (-0.89)	-2.02∗
Experimental Group	5.28 (0.11)		

(SE) Standard error of mean, ∗∗∗p < 0.001, ∗∗p < 0.01, ∗p < 0.05.

Table 5 shows that the *p-value* was less than 0.05, which means that there is a statistically significant difference in the performance of the two groups. Compared to the control (traditional learning) group, the experimental (YouTube) group reflected better but still modest improvement.

The analysis above shows that there is significant progress in the speaking performance of the experimental group. To determine which of the four constructs under investigation were most noticeably advanced among the experimental group, One-Way ANOVA Test was used (Table 6).

**Table 6:** Results of one-way ANOVA test for the experimental group.

Construct	Test	M (SE)	Construct Rank by Progress	F value
Fluency & coherence	Pre-Test	4.73 (0.18)	2	3.728∗
	Post-Test	5.33 (0.17)		
Lexical resource	Pre-Test	5.33 (0.18)	3	
	Post-Test	5.80 (0.14)		
Grammatical Range and Accuracy	Pre-Test	5.38 (0.17)	4	
	Post-Test	5.80 (0.17)		
Pronunciation	Pre-Test	4.38 (0.17)	1	
	Post-Test	5.10 (0.16)		

(SE) standard error of mean, ∗∗∗p < 0.001, ∗∗p < 0.01, ∗p < 0.05.

Table 6 shows that there is a significant difference in the performance of the experimental group's students in the four descriptors since the F value is greater than 2 at a significance level of 0.05.

The sub-scale that improved the most in the speaking skill of the experimental group is ‘pronunciation’ with a mean difference of (0.72), followed by ‘fluency & coherence’ with a mean difference of (0.6). ‘Lexical resource’ and ‘grammatical range and accuracy’ ranked third and fourth, respectively, with a mean difference of (0.47) and (0.42) respectively.

## 5 Discussion of results

This study aimed to examine the development of the speaking skill descriptors among the experimental group subjects subsequent to the treatment they were subjected to in YouTube. The differences between the pre-test and post-test scores showed that students’ speaking skill performance was higher for this group than their counterparts in the control group. This is consistent with Silviyanti (2014), who affirmed the unquestionable value of using YouTube in EFL classrooms, where students get to be eager and keen to watch YouTube videos and other videos in the target language. This, of course, provides them with an opportunity to practice the speaking skill interactively and communicatively.

The results of this study are in agreement with Omer (2017), who showed that YouTube improved EFL students’ listening and speaking skills and encouraged them to communicate verbally in English. Furthermore, YouTube acquainted students with native English speakers' culture, which, in turn, enhanced their language proficiency.

Many researchers affirmed the benefit and usefulness of YouTube in teaching speaking skills to EFL students. A recent study conducted by Syafiq et al. (2021, p. 50) showed that “YouTube video as English learning material improved the speaking skill of students including fluency, vocabulary, pronunciation, grammar, and content.” This ties in well with the results reported in the current study.

There are several benefits associated with using video clips to teach a foreign language. Nasution (2019, p. 31) believes that “YouTube may present language teachers with a useable medium for introducing language points, finding topics for discussion, or examples of authentic English, with proper guidance and suggested videos available on the site.” The variability of YouTube holds here as students in the experimental group had the opportunity to watch videos on various topics and situations, which enhanced their speaking skills. In addition, the eagerness and enthusiasm of the experimental group students were evident during student question and answer sessions and topic discussion. In line with this, Silviyanti (2014) reported that using YouTube in the EFL classroom seems compelling, encouraging, and beneficial, where students appear to be enthusiastic and willing to watch different videos, then practice pronunciation and speaking in the same way as native speakers.

The research findings reported by Kurniawan (2019, p. 324) showed that “students consider that the YouTube can help their speaking skill becoming much better.” In the same vein, research done by Meinawati et al. (2020, p. 1) on ‘increasing English speaking skills through YouTube’ revealed that “the result of using YouTube was very effective because it allowed students to speak with more confidence and expression.” The findings in these two studies are consistent with the findings of this study.

A recent study conducted by Syafiq et al. (2021, p. 50) showed that “YouTube video as English learning material improved speaking skill of students including fluency, vocabulary, pronunciation, grammar, and content.” This as well is in agreement with the findings of this piece of research.

The results reported by Jati et al. (2019, p. 101) showed that “the students' speaking skill was improving in three aspects 1) accuracy, 2) fluency, 3) performance”. Similar findings on the effectiveness of YouTube videos in improving students’ speaking skills were reported by different scholars (see Albahlal, 2019; Arroyyani, 2018; Pratama et al., 2020; Setiawan and Wiedarti, 2020; Tristiana and Swondo, 2020).

The present study, which is the beginning of a series of empirical research endeavors, is limited in focus. It addresses one specific level of the EFL students’ skills, namely speaking. In addition, the study sampled a relatively small number of university students in one higher education institution in Jordan to set the stage for additional research attempts on the other components of the IELTS speaking band which consists of four main categories, namely fluency & coherence, lexical resource, grammatical range & accuracy, and pronunciation. Once the research base is expanded and intensified with broader coverage, the future research findings will lend support to the results of the current study and will subsequently have more local, regional, and global currency and dissemination.

## 6 Conclusions and recommendations

Ever since it was launched in 2005, the YouTube video-sharing platform has made giant strides in revolutionizing sources of information and knowledge base interactively. The current study subscribes to this thesis by investigating the impact of YouTube videos in EFL speaking classes. Our research findings have most succinctly shown that not only does the use of YouTube in the EFL classroom help boost the speaking skill, but it also develops fluency & coherence through enhancing pronunciation, lexical choices, and grammatical structures, leading to a coherent and meaningful discourse.

Inhibitions of speaking, debating, and exchanging opinions diminish shortly after experimenting with the YouTube materials, which trigger spontaneous students' responses reflecting better pronunciation, more careful use of vocabulary, with reasonable fluency & coherence. Based on statistical analyses, these pieces of evidence showed statistically significant differences in the experimental group's scores following their YouTube experience. The results have shown a direct interrelationship between students' use of YouTube videos to improve their speaking, coherence and oral fluency, and lexical choices. Based on students' scores before and after integrating the YouTube media, the results showed statistically significant differences in favor of the post-YouTube phase. Compared to the experimental groups' progress throughout the semester, the control group scores and means were noticeably lower.

The current research findings are in line with the surging trend of integrating educational technology in the classroom following calls for transitioning to blended and full-fledged online learning. There is no better example of the compelling need for a swift shift in this direction than the COVID-19 crisis which affected all spheres of life (Al-Salman and Haider, 2021a). COVID-19 has revolutionized and reshaped the educational paradigm internationally (Al-Salman and Haider, 2021b; Haider and Al-Salman, 2020a). In parallel to this reality, top-notch and state-of-the-art multimedia educational technology tools, top among which is audiovisuals, should be abreast of innovations in the teaching-learning operation. To this end, we recommend that YouTube videos, digital technology, and multimedia packages be an integral part of the educational syllabi. Consequently, further research is needed to create new EFL curricula and syllabi based on innovations in education technology and their applications for academic purposes.

Given the limitations of the current study which addresses the speaking skill per se, and the positive effects and takeaways realized in the students' performance in spoken English, we strongly recommend that more empirical research endeavors be conducted to probing into the effect of YouTube videos in promoting Jordanian EFL students' level of achievement in the other three language skills, namely listening, reading and writing. The findings of such potential research endeavors in an e-learning environment will benefit from research findings reported in the literature review section, especially research contributed by researchers (see Jati et al., 2019; Martin et al., 2020; Nasution, 2019; Pratama et al., 2020; Riswandi, 2016; Syafiq et al., 2021). Such a call for further rigorous research in this particular domain is inspired by the present study's findings, which recommend that YouTube videos be embedded into the EFL classroom to improve students' speaking skills.

## Declarations

### Author contribution statement

Hadeel A Saed: Conceived and designed the experiments; Analyzed and interpreted the data; Wrote the paper.

Ahmad S Haider: Performed the experiments; Analyzed and interpreted the data; Wrote the paper.

Saleh Al-Salman, Riyad F. Hussein: Performed the experiments; Analyzed and interpreted the data; Contributed reagents, materials, analysis tools or data; Wrote the paper.

### Funding statement

This research did not receive any specific grant from funding agencies in the public, commercial, or not-for-profit sectors.

### Data availability statement

Data will be made available on request.

### Declaration of interests statement

The authors declare no conflict of interest.

### Additional information

No additional information is available for this paper.
