# Supplementary material for: The use of YouTube in developing the speaking skills of Jordanian EFL university students
Source: Heliyon. 2021 Jul 12;7(7):e07543. doi: 10.1016/j.heliyon.2021.e07543 (PMC8287226; doi:10.1016/j.heliyon.2021.e07543)

**Appendix 1:** IELTS speaking band descriptors (<https://www.ielts.org/-/media/pdfs/speaking-band-descriptors.ashx?la=en>)


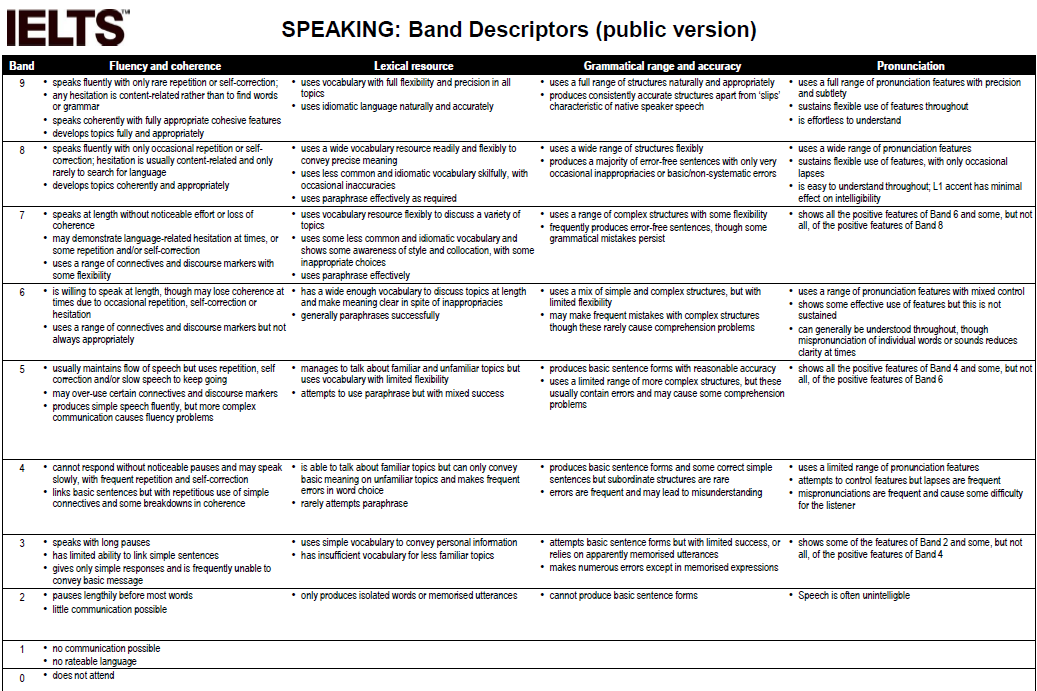

Supplement: Appendix 1 [file mmc1.docx]
